# Increased Curie Temperature Induced by Orbital Ordering in La_0.67_Sr_0.33_MnO_3_/BaTiO_3_ Superlattices

**DOI:** 10.1186/s11671-018-2441-5

**Published:** 2018-01-17

**Authors:** Fei Zhang, Biao Wu, Guowei Zhou, Zhi-Yong Quan, Xiao-Hong Xu

**Affiliations:** 1 Key Laboratory of Magnetic Molecules and Magnetic Information Materials of Ministry of Education, School of Chemistry and Materials Science, Shanxi Normal University, Linfen, 041004 China; 2 Research Institute of Materials Science, Shanxi Normal University, Linfen, 041004 China; 3 Suzhou Institute of Nano-Tech and Nano-Bionics, China Academy of Sciences, Suzhou, 215123 China

**Keywords:** La_0.67_Sr_0.33_MnO_3_/BaTiO_3_ superlattices, High Curie temperature, Orbital ordering, Tensile strain, 75.47.Lx, 51.60.+a, 68.35.Ct

## Abstract

Recent theoretical studies indicated that the Curie temperature of perovskite manganite thin films can be increased by more than an order of magnitude by applying appropriate interfacial strain to control orbital ordering. In this work, we demonstrate that the regular intercalation of BaTiO_3_ layers between La_0.67_Sr_0.33_MnO_3_ layers effectively enhances ferromagnetic order and increases the Curie temperature of La_0.67_Sr_0.33_MnO_3_/BaTiO_3_ superlattices. The preferential orbital occupancy of e_g_(*x*^*2*^*–y*^*2*^) in La_0.67_Sr_0.33_MnO_3_ layers induced by the tensile strain of BaTiO_3_ layers is identified by X-ray linear dichroism measurements. Our results reveal that controlling orbital ordering can effectively improve the Curie temperature of La_0.67_Sr_0.33_MnO_3_ films and that in-plane orbital occupancy is beneficial to the double exchange ferromagnetic coupling of thin-film samples. These findings create new opportunities for the design and control of magnetism in artificial structures and pave the way to a variety of novel magnetoelectronic applications that operate far above room temperature.

## Background

A common observation in perovskite manganite films is that the Curie temperature (*T*_C_) decreases with the reduction of film thickness, which limits their potential for spintronic devices such as field-effect transistors, magnetic tunnel junctions, spin valves, and nonvolatile magnetic memory [1–5]. This is the so-called “dead layer,” defined as the thinnest layer for which ferromagnetic behavior is observed [6–8]. This dead layer phenomenon may be related to electronic and/or chemical phase separation [9, 10], to growth characteristics and microstructure [11, 12], or to manganese e_g_ orbital reconstruction [13, 14]. Recently, many efforts have been made to increase the *T*_C_ of ultrathin perovskite manganite films by superlattice interface control and precise strain tuning [15–18]. Among the perovskite manganites, La_0.67_Sr_0.33_MnO_3_ (LSMO) films have drawn increasing interest due to their colossal magnetoresistance effect, high *T*_C_, and half metallicity [19–23]. Also LSMO-based heterostructures have been investigated because of the interfacial couplings and intermixing of atoms etc. [24–28]. M. Ziese et al. reported ferromagnetic order of ultrathin LSMO layers in LSMO/SrRuO_3_ superlattices stabilized down to layer thicknesses of at least two unit cells (u.c.) that exhibits a *T*_C_ above room temperature [29]. First principle calculations indicate that the *T*_C_ of LSMO films can be increased by more than an order of magnitude by controlling orbital ordering using the regular intercalation of adequate layers in LSMO/BaTiO_3_(BTO) superlattices. In such a configuration, the LSMO layers with occupied e_g_(*x*^*2*^*–y*^*2*^) orbitals are associated with a strong in-plane double exchange, resulting in a high *T*_C_ [30]. This phenomenon has been observed in temperature-dependent magnetization data [30].

In this work, we synthesized LSMO/BTO superlattices using pulsed laser deposition (PLD) and reveal the relationship between the origin of high *T*_C_ and manganese e_g_ orbital occupancy through the use of X-ray linear dichroism (XLD) measurements. We show that the regular intercalation of BTO layers between LSMO layers can effectively enhance ferromagnetic order and increases the *T*_C_ of ultrathin LSMO films due to the orbital occupation of e_g_(*x*^*2*^*–y*^*2*^) in Mn^3+^ ions. Notably, the origin of the *T*_C_ increase is different from the one suggested theoretically by A. Sadoc et al., who showed that only the central LSMO layers contribute to high *T*_C_ and that the interfacial layers adjacent to the BTO layers are associated with a weak in-plane double exchange due to e_g_(*3z*^*2*^*–r*^*2*^) orbital occupation [30]. We find that the preferential orbital occupancy of e_g_(*x*^*2*^*–y*^*2*^) in both of the central and the interfacial LSMO layers is induced by BTO layer strain, and gives rise to the in-plane double exchange coupling in LSMO/BTO superlattices, resulting in high *T*_C_. Our findings provide a method to design and control magnetism in artificial structures and have potential for spintronic device applications—including spin-valve devices or nonvolatile magnetic memory working at temperatures far above room temperature.

## Methods

**Fig. 1:**
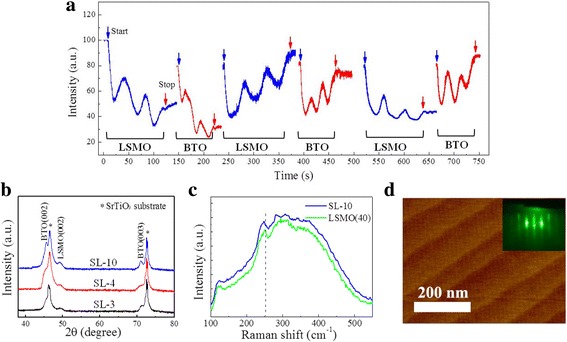
**a** RHEED intensity oscillations for the growth of the SL-3 sample. **b** XRD patterns for three different SL-n samples (*n* = 3, 4, 10). **c** Raman spectra for the SL-10 and LSMO(40) samples measured at 300 K. **d** AFM image of a BHF-etched, bare (001) SrTiO_3_ substrate. The inset shows the RHEED diffraction pattern of the SL-3 sample

(001)-oriented [(LSMO)_3_/(BTO)_3_]_*n*_ superlattice (denoted as SL-n, where 3 is the number of unit cells, *n* = 3, 4, 10 is the number of cycles) samples were synthesized on (001) SrTiO_3_ substrates using PLD. A stoichiometric polycrystalline target was used in a 100-mTorr oxygen environment at a substrate temperature of 725 and 780 °C for LSMO and BTO, respectively. A KrF excimer laser (*λ* = 248 nm) with a 2 Hz repetition rate was employed. Energy of 350 and 300 mJ was focused on the targets to obtain the LSMO and BTO layers, respectively. After the growth, the samples were annealed in a 300-Torr oxygen atmosphere in situ for 1 h to improve their quality and reduce their inherent oxygen deficit and then cooled to room temperature. As a reference, two LSMO films with 3 and 40 u.c. thickness (denoted as LSMO(3) and LSMO(40), respectively) were also prepared using PLD under the same conditions for comparison with the SL-n superlattices. To grow films epitaxially with atomic precision, we prepared an atomically flat, single-terminated SrTiO_3_ surface by etching in an NH_4_F-buffered HF solution (BHF) and subsequently annealing in an oxygen atmosphere at a temperature of 960 °C. The surface topography of a BHF-treated, bare (001) SrTiO_3_ substrate was characterized by atomic force microscopy (AFM) analysis, as shown in Fig. 1d. The surface is very smooth, and there are clear steps separating the terraces.

The growth process for each film was monitored in situ using real-time reflection high-energy electron diffraction (RHEED) analysis, providing precise control of the thickness at the unit cell scale and an accurate characterization of the growth dynamics. The crystal structures and surface morphologies were investigated using X-ray diffraction (XRD) and transmission electron microscopy (TEM). To confirm the strain in the samples, Raman spectra were also recorded using a microscopic confocal Raman spectrometer (RM2000, Renishaw, England) excited with a 514.5 nm Ar^+^ ion laser. The magnetic properties and *T*_C_ of the samples were measured with a superconducting quantum interference device (SQUID) magnetometer with in-plane applied magnetic field. The magnetization was calculated after a linear background subtraction of the SrTiO_3_ substrate diamagnetic contribution. The transport properties were determined in the Van der Pauw four-point probe configuration using a Quantum Design Physical Properties Measurement System (PPMS) over temperatures ranging from 20 to 365 K. X-ray absorption spectroscopy (XAS) and XLD measurements were made at Beamline BL08U1A of the Shanghai Synchrotron Radiation Facility and U19 of National Synchrotron Radiation Laboratory in the total electron yield (TEY) mode at room temperature.

## Results and Discussion

**Fig. 2:**
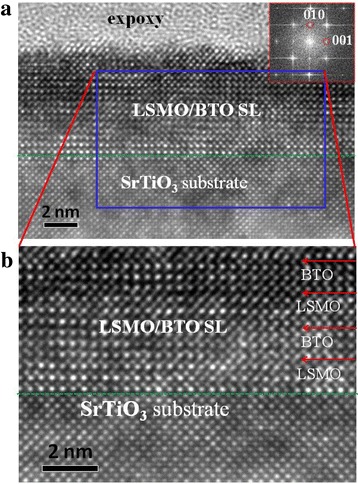
**a** A cross-section HRTEM image of the SL-3 sample. The inset shows the corresponding FFT patterns. **b** The enlarged blue rectangle drawing with the interfaces between the LSMO and BTO layers indicated by red arrows

Figure 1a shows the RHEED oscillations recorded during the growth of the SL-3 sample on a TiO_2_-terminated (001) SrTiO_3_ substrate. The LSMO and BTO film thicknesses were controlled by counting the RHEED intensity oscillations. For optimized conditions, RHEED oscillations remain visible throughout the superlattice deposition process, indicating a layer-by-layer growth. The inset of Fig. 1d shows the clear streaky RHEED diffraction pattern after the growth of the SL-3 sample. Typical XRD patterns shown in Fig. 1b reveal high-quality growth in the (001) orientation for all three superlattices. As expected, the LSMO peaks shift slightly to a higher angle while the BTO peaks shift to a lower angle (compared to the bulk value), which reflects the strain state of the interfaces between the LSMO layers and the BTO layers (i.e., in-place cell parameter elongation for LSMO and reduction for BTO). This desired strain can be maintained over the whole film thickness due to the repeating intercalation of LSMO and BTO layers. Raman spectra measured at 300 K for the SL-10 and LSMO(40) samples are shown in Fig. 1c. Compared to LSMO(40) sample, a slight low-frequency shift of bands at 252 cm^− 1^ was observed in SL-10 sample, indicating the LSMO layers in SL-10 sample with a tensile strain induced by BTO layers [31–33]. In addition, the high quality of the superlattices was confirmed by TEM. Figure 2a is the cross-sectional high-resolution TEM (HRTEM) of the SL-3 sample on a (001)-oriented SrTiO_3_ substrate, endorsing high-quality epitaxial growth of LSMO/BTO superlattice. The inset of Fig. 2a is the corresponding fast Fourier transform (FFT), suggesting that the film is indeed in single phase. Figure 2b shows the enlarged image of Fig. 2a. The image shows atomically sharp interfaces between the LSMO and BTO layers highlighted by red arrows. In the superlattices, there is no obvious interdiffusion at the interfaces, and the LSMO and BTO layers are fully strained to the SrTiO_3_ substrates. This observation was consistent with the XRD results.

**Fig. 3:**
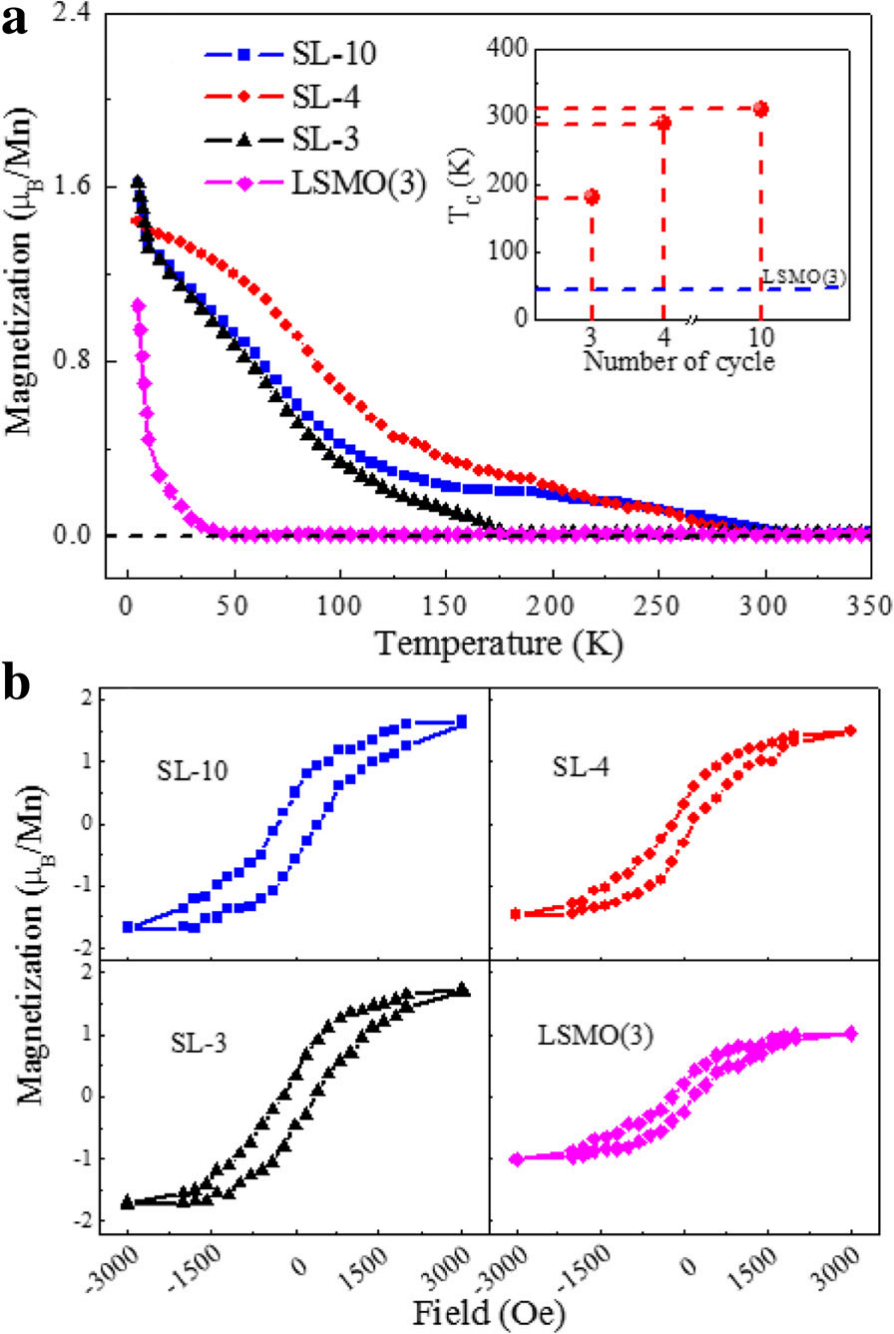
**a** Temperature-dependent magnetization of different SL-n samples (*n* = 3, 4, 10) and an ultrathin LSMO film with a 3-u.c. thickness. The magnetic field of 3000 Oe was applied in-plane along the SrTiO_3_ substrates. The inset shows cycle number dependence on the *T*_C_. **b** The corresponding magnetic hysteresis loops of four samples measured at 5 K

Next, we present a description of the magnetic properties of the SL-n samples. The temperature-dependent magnetization for SL-n films with *n* = 3, 4, 10, as well as the LSMO(3) sample, are shown in Fig. 3a. Here, the measurement is carried out over a temperature range from 5 to 350 K with a magnetic field (3000 Oe) applied parallel to the surface of the SrTiO_3_ substrates. Note that the *T*_C_ of the superlattices is significantly improved compared to the LSMO(3) film [6], of which *T*_C_ is around 45 K (see the inset in Fig. 3a). For the SL-10 sample, the *T*_C_ increases above 265 K compared to the LSMO(3) film and reaches a maximum value of *T*_C_~310 K. Figure 3b shows corresponding magnetic hysteresis loops for the four samples measured at 5 K, showing obvious ferromagnetic signal with a saturation magnetization (*Ms*) of ~ 1.5 μ_B_/Mn—except for the LSMO(3) film. Here, the ferromagnetism of the LSMO layers in the SL-n samples comes from the total LSMO triple layers, which is different from those reported by A. Sadoc et al., who showed that the ferromagnetic exchange is just related to the central LSMO layers and is independent of the interfacial LSMO layers adjacent to BTO layers using first principle calculations [30]. Given that ferromagnetism is only derived from the central LSMO layers, the *M*_s_ value of our SL-n films calculated from the original measurement data will become ~ 4.5 μ_B_/Mn, which will exceed the theoretical low temperature value of the LSMO (~ 3.67 μ_B_/Mn) [34]. Note that the *M*_s_ per spin is much less than of bulk LSMO, suggesting either a fraction of nonmagnetic spins, a ferrimagnetic spin arrangement, or strong spin-canting [18, 35]. More work will be needed to quantify decreased *M*_s_ in this LSMO/BTO system. Also, the magnetic anisotropy of the SL-n samples with *n* = 3, 4, 10 were studied. The magnetic hysteresis loops for the magnetic field applied in-plane and out-of-plane measured at 5 K (not shown here) display that the easy magnetization axis for the three samples is parallel to the film plane direction, which is related to the orbital occupancy in LSMO layers, as discussed below.

We now focus on the correlation between increased *T*_C_ and electron orbital occupancy in the LSMO/BTO superlattices. It is known that the Mn^3+^ ions are Jahn-Teller active, and a slightly distorted orthorhombic structure can stabilize one of the e_g_ orbitals. Supposing the e_g_(*3z*^*2*^*–r*^*2*^) is occupied, an interlayer double exchange interaction between the Mn^3+^ and Mn^4+^ ions will take place primarily along the *c* direction for (001)-oriented LSMO material. When e_g_(*x*^*2*^*–y*^*2*^) is occupied, the intralayer double exchange will become very strong and the interlayer double exchange will decline in strength. In ultrathin films, in-plane interactions dominate the magnetic exchange and *T*_C_. Thus, control of the orbital ordering is important for obtaining high-temperature ferromagnetism. That is to say, a high-occupancy probability of the e_g_(*x*^*2*^*–y*^*2*^) orbital can result in a high *T*_C_ for (001)-oriented LSMO films.

**Fig. 4:**
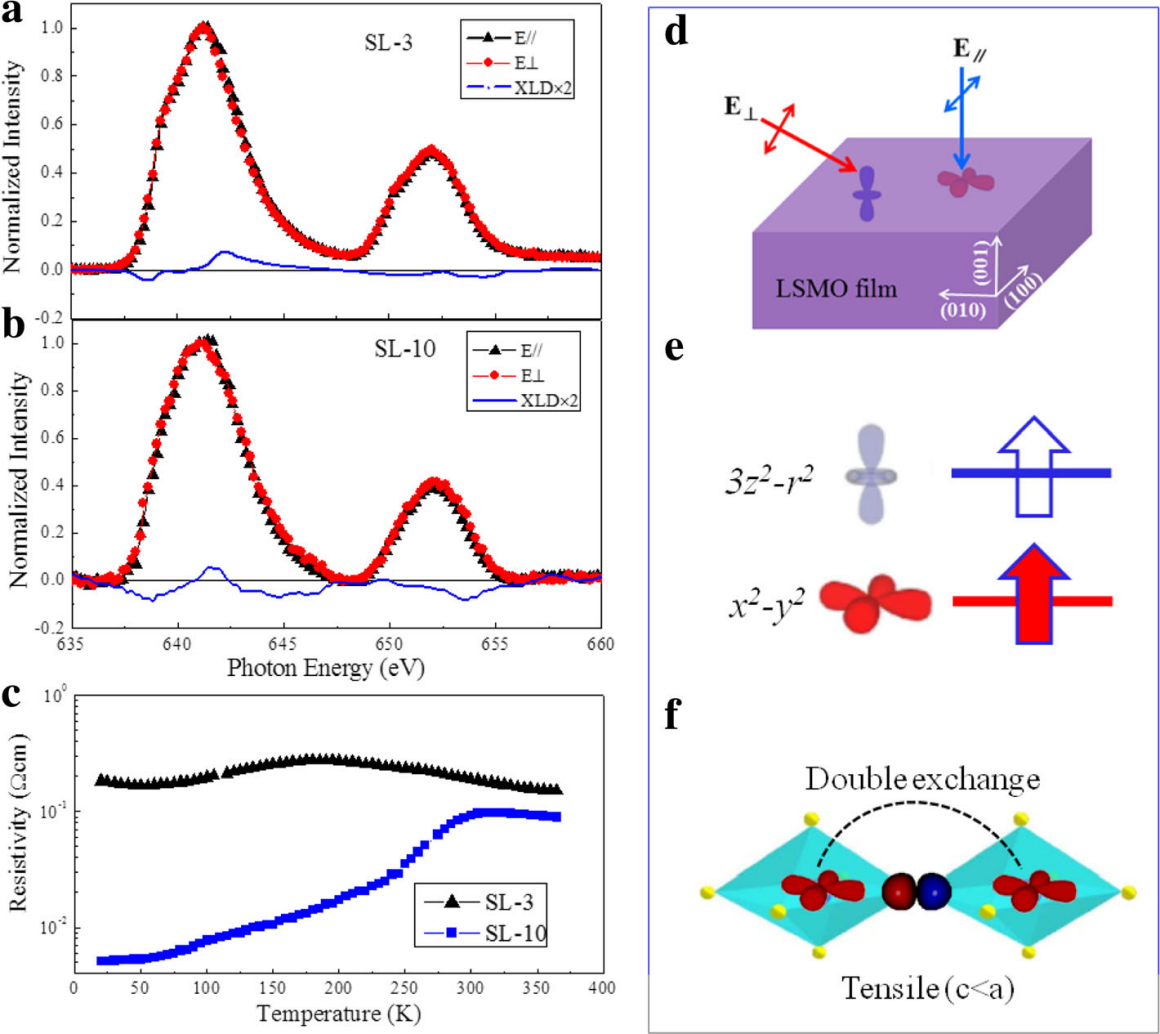
**a, b** Normalized XAS and XLD curves for samples SL-3 and SL-10 measured at room temperature. **c** Temperature-dependent resistivity measured in the temperature range from 20 to 365 K for (001)-oriented SL-n samples where *n* = 3 and 10. **d** Experimental configuration schematic diagram for XAS measurements with different X-ray incident angles. **e** Schematic representation of the electronic orbital occupancy of manganese e_g_ in (001)-oriented LSMO/BTO superlattices. **f** Proposed double exchange coupling mechanism along the in-plane direction

In our LSMO/BTO samples, the lattice parameter of the BTO (*a* = 0.397–0.403 nm from a tetragonal to rhombohedral phase) is larger than that of LSMO (*a* = 0.387 nm), resulting in a ~ 4% lattice mismatch [36–38]. Thus, the LSMO layers in our superlattices are in a high-tensile strain state (*c* < a), causing occupancy in the e_g_(*x*^*2*^*–y*^*2*^) orbital [39]. We now discuss the manganese e_g_ orbital occupancy in relation to XLD measurements, which is an extremely sensitive probe for the electronic structure and the d orbital (e_g_) electron occupancy (schematic diagram shown in Fig. 4d), which has proven in referential occupancy at interfaces [14]. The XAS spectra were measured at the Mn L_2,3_-edges for the photon polarization (E) parallel to the sample plane (E_//_) and perpendicular to it (E_⊥_). The XLD is calculated as the XAS intensity difference between the E_//_ and E_⊥_ components to determine the occupancy of the Mn^3+^ e_g_ orbitals. In (001)-oriented LSMO films, the out-of-plane direction corresponds to [001], and the in-plane direction was obtained with E//[100], as shown in Fig. 4d. The area under the XLD curve at the L_2_-edge peak (ΔXLD) represents the difference between the relative occupancies of the e_g_(*x*^*2*^ *− y*^*2*^*/3z*^*2*^ *− r*^*2*^) orbitals. A positive/negative ΔXLD (on average) is ascribed attributed to a preferential occupancy of the e_g_(*3z*^*2*^ *− r*^*2*^*)/(x*^*2*^ *− y*^*2*^) orbitals for (001) LSMO films. Figure 4a, b shows the XLD spectra, as well as the in-plane and out-of-plane XAS spectra, of SL-3 and SL-10 samples. The ΔXLD area at the L_2_-edge peak is negative, implying a preferential occupancy of the e_g_(*x*^*2*^*–y*^*2*^) orbital (see Fig. 4e), which is consistent with the results reported by D. Pesquera et al. [39]. Consequently, in our LSMO/BTO superlattices, the interfacial tensile strain is originated from the lattice mismatch between the BTO and LSMO layers. It induces in-plane orbital ordering of the e_g_(*x*^*2*^*–y*^*2*^) orbital occupancy in the LSMO layers, achieving high *T*_C_. This negative value of the ΔXLD area is also evidence that the Mn^3+^ ions in the LSMO triple layers have the same orbital occupancy, which contributes to high-temperature ferromagnetism. Also, the absolute value of ΔXLD for the SL-10 sample is significantly larger than that of the SL-3 sample, which corresponds to the increased *T*_C_ seen in Fig. 3a.

Figure 4c shows temperature-dependent resistivity in the temperature range from 20 to 365 K for (001)-oriented SL-n superlattices with *n* = 3 and 10, respectively. The two samples exhibit a metal-to-insulator transition temperature (*T*_MI_). The *T*_MI_ values of 178 and 310 K for samples SL-3 and SL-10, respectively, correspond to the *T*_C_ shown in Fig. 3a. This supports the scenario for a transition at *T*_C_ from a paramagnetic insulating phase to a ferromagnetic metallic phase. Thus, the high-temperature ferromagnetism originates from in-plane double exchange interactions between the Mn^3+^ and Mn^4+^ ions as shown in Fig. 4f [40, 41]. In-plane overlap between (partly filled) Mn e_g_(*x*^*2*^*–y*^*2*^) with O 2*p*_*x*_ and O 2*p*_*y*_ creates stronger ferromagnetic coupling than that between (more empty) Mn e_g_(*3z*^*2*^*–r*^*2*^).

## Conclusions

In summary, LSMO/BTO superlattices were prepared using PLD and the relationship between high *T*_C_ and manganese e_g_ orbital occupancy was revealed combined with XLD spectra. We showed that the regular intercalation of BTO layers between LSMO layers effectively enhances ferromagnetic order and increases the *T*_C_ of LSMO/BTO superlattices. The preferential orbital occupancy of e_g_(*x*^*2*^*–y*^*2*^) in LSMO layers induced by tensile strain of BTO layers is beneficial to the in-plane double exchange ferromagnetic coupling between Mn^3+^ and Mn^4+^ ions, resulting in a large *T*_C_. Our findings create new opportunities for the design and control of magnetism in artificial structures and offer considerable potential for applications in novel magnetoelectronic applications, including nonvolatile magnetic memory working far above room temperature.

## Abbreviations

AFM: Atomic force microscopy
BHF: NH_4_F-buffered HF solution
FFT: Fast Fourier transform
*M*_s_: Saturation magnetization
PLD: Pulsed laser deposition
PPMS: Physical properties measurement system
RHEED: Real-time reflection high-energy electron diffraction
SQUID: Superconducting quantum interference device
*T*_C_: Curie temperature
TEM: Transmission electron microscopy
TEY: Total electron yield
*T*_MI_: Metal-to-insulator transition temperature
XAS: X-ray absorption spectroscopy
XLD: X-ray linear dichroism
XRD: X-ray diffraction
